# Psychosis and Pericardial Effusion in Neuropsychiatric Systemic Lupus Erythematosus: Diagnostic Challenges in a Language-Discordant Encounter

**DOI:** 10.7759/cureus.116002

**Published:** 2026-09-09

**Authors:** Beylul Negassi, Gabriel Cruz, Ivory N Jean-Paul, Yazan Nagi, Alan Tusher

**Affiliations:** 1 Medical School, Downstate Health Sciences University, Brooklyn, USA; 2 Medical School, Donald and Barbara Zucker School of Medicine at Hofstra/Northwell, Hempstead, USA; 3 Psychiatry, Kings County Hospital Center, Brooklyn, USA

**Keywords:** collateral history, diagnostic delay, health care disparities, interdisciplinary collaboration, language barriers in healthcare, neuroimaging, neuropsychiatric systemic lupus erythematosus (npsle), psychiatric manifestations, psychosis, systemic lupus erythematosus

## Abstract

Neuropsychiatric systemic lupus erythematosus (NPSLE) is a diagnostically complex manifestation of systemic lupus erythematosus (SLE) that may initially present with significant psychiatric symptoms, contributing to delayed recognition and treatment. We describe a 36-year-old non-English-speaking female patient with a recent history of intermittent medical and psychiatric encounters over 1 year, during which progressive psychosis was attributed to a primary psychiatric illness. Her course was characterized by fragmented care across institutions, limited collateral history, language discordance, and diagnostic anchoring on psychiatric diagnosis despite evolving systemic findings.

Several weeks after surgical management of a large pericardial effusion, the patient presented to the emergency department with acute psychiatric decompensation marked by hallucinations and behavioral abnormalities. She was admitted for cardiac monitoring and treated for presumed primary psychosis. Subsequent multidisciplinary reassessment, delayed acquisition of outside records, and neuroimaging revealing diffuse parenchymal enhancement raised concern for NPSLE. Once lupus-directed immunosuppressive treatment was initiated, the patient demonstrated substantial improvement in psychiatric symptoms.

This vignette highlights the diagnostic challenges of autoimmune disease presenting with psychiatric manifestations and underscores the importance of systematic collateral assessment, cross-institutional communication, and interdisciplinary collaboration in reducing diagnostic delay.

## Introduction

Neuropsychiatric systemic lupus erythematosus (NPSLE) is a diagnostically challenging manifestation of autoimmune disease that can closely mimic primary psychiatric illness, often leading to misattribution and delays in appropriate treatment. The condition encompasses a broad spectrum of neurological and psychiatric presentations, with symptoms that may emerge early yet remain difficult to recognize [1]. The absence of pathognomonic findings, the need to exclude numerous alternative etiologies, and heavy reliance on clinical judgment further complicate timely diagnosis [2]. No single clinical, serologic, cerebrospinal fluid, or neuroimaging finding establishes NPSLE; diagnosis instead relies on exclusion of competing causes and integration of the neuropsychiatric presentation with evidence of underlying systemic lupus erythematosus (SLE) [1,2]. New or atypical psychiatric symptoms occurring alongside cognitive or neurological abnormalities or evidence of systemic lupus activity should prompt consideration of NPSLE in the differential diagnosis [1].

Challenges in making the diagnosis of NPSLE are amplified in the presence of social and structural barriers, such as language discordance, fragmented care, and limited access to collateral information, all of which can obscure clinical insight and continuity [3,4]. The diagnostic heterogeneity of NPSLE is also reflected in widely varying prevalence estimates, depending on case definition and attribution methodology [5,6]. This case illustrates how the intersection of clinical complexity and systems-level barriers can prolong diagnostic uncertainty, while underscoring the critical role of interdisciplinary collaboration and systematic collateral assessment in achieving diagnostic clarity.

## Case presentation

A 36-year-old woman with limited English proficiency, a chart diagnosis of schizophrenia, and a psychiatric hospitalization approximately 1 year earlier was brought to the emergency department for several days of worsening behavioral and psychiatric symptoms. Her prior psychiatric admission had immediately followed a prolonged medical hospitalization for sepsis secondary to right-elbow arthritis. The available records did not establish a continuous course of psychotic symptoms between the earlier admission and the current presentation. 

Two weeks before the current admission, the patient was surgically managed for a large pericardial effusion at an outside institution. Records obtained later during the hospitalization showed that SLE had been recognized during an outside admission a year prior, based on a high antinuclear antibody titer, positive anti-Sm and anti-RNP antibodies, and marked hypocomplementemia. This information was not available to the treating team throughout this patient’s hospital course.

During the several days preceding admission, her husband reported sleep disturbance, talking to herself, hallucinations, physical aggression toward him, failure to recognize her son, and episodes of leaving the home, which raised concerns regarding her safety. He described similar behavior during a prior episode but considered the current deterioration more severe. He also reported that she had not taken her prescribed psychiatric medication for an uncertain period. A possible suicidal statement was reported on the day before admission, although no plan was documented and the patient was unable to participate reliably in a comprehensive risk assessment. The available documentation did not characterize the reported hallucinations by sensory modality, content, frequency, or command quality, and no specific fixed delusional belief was recorded.

On arrival to the psychiatric emergency service, she appeared disheveled, stood staring into space, intermittently mumbled to herself, and was minimally able to engage despite interpreter-assisted assessment. During subsequent evaluations, a medical interpreter was used, although the patient, at times, did not respond to questions presented through the interpreter. Her husband was contacted separately for collateral history and was not documented as serving as an interpreter. He provided information regarding her recent behavioral changes but was unaware of her prior lupus or rheumatologic diagnosis. She repeatedly stated, “There is an emergency,” without further explanation. She received risperidone 2 mg and lorazepam 2 mg orally for disorganized behavior. Because her heart rate was approximately 145 beats/min and she had recently undergone a cardiac procedure, she was transferred to the medical emergency department before psychiatric clearance.

In the medical emergency department, she was alert but agitated, unable to answer questions appropriately, and inconsistently oriented to approximately zero or one domain. Point-of-care glucose was initially in the 30-mg/dL range and increased to the 160-mg/dL range after intravenous dextrose. She required olanzapine 5 mg intramuscularly and midazolam 4 mg intramuscularly to facilitate medical evaluation. Tachycardia improved with intravenous fluids. The subsequent course included hypotension requiring brief vasopressor treatment and admission to the intensive care unit. She remained afebrile, and no nuchal rigidity or rash was documented. Initial hypoglycemia and hemodynamic instability were recognized as possible contributors to her altered mental status. However, a standardized delirium assessment, such as the Confusion Assessment Method or 4AT, was not documented.

Following medical stabilization, prominent psychiatric and cognitive abnormalities persisted. During neuropsychiatric assessment, the patient was initially calm and cooperative but became increasingly irritable, emotionally labile, tearful, and demanding as the interview progressed. Her thought process was perseverative, illogical, intermittently disorganized, and at times marked by thought blocking. She required repeated attempts at re-engagement and appeared to confabulate the extent of her medication adherence and cooperation during the hospitalization. Flattened affect and religious preoccupation were also documented.

Cognitive findings included difficulty recalling details of her employment, the location of a church she reportedly attended regularly, her medical history, and a recent conversation concerning the lupus diagnosis. She was unable to explain the reason for hospitalization or restate the medical information that had just been provided to her. Insight was poor, and her ability to appreciate the need for hospitalization and treatment was impaired. Although irritability, lability, and tearfulness were observed, no sustained manic syndrome or major depressive episode was documented. She had no documented symptoms of catatonia or neurological symptoms such as seizures, focal neurological deficits, abnormal involuntary movement, or meningeal signs.

Because the psychiatric and cognitive abnormalities persisted after correction of hypoglycemia and hemodynamic instability, the differential diagnosis was broadened. Laboratory testing demonstrated an antinuclear antibody titer of 1:1280; anti-Sm, anti-RNP, anti-SSA/Ro, and anti-chromatin antibody levels greater than 8; decreased complement levels, with C3 of 72 mg/dL and C4 of 6 mg/dL; and an elevated erythrocyte sedimentation rate of 53 mm/hr. Anti-double-stranded DNA (anti-dsDNA) was within the laboratory reference range. The normal anti-dsDNA result did not independently establish or exclude neuropsychiatric involvement (Table 1).

**Table 1:** Autoimmune serologic and inflammatory markers Autoimmune and inflammatory markers demonstrated a high antinuclear antibody (ANA) titer and positive extractable nuclear antigen antibodies (anti-Sm, anti-RNP, anti-SSA/Ro, and anti-chromatin). Anti–double-stranded DNA (anti-dsDNA), anti-ribosomal P, and DRVVT (Lupus anticoagulant; dilute Russell's viper venom time) screening/confirmatory ratios were within normal limits. Complement levels were decreased (C3 and C4), and the erythrocyte sedimentation rate (ESR) was elevated. Values are presented with corresponding reference ranges; ANA is reported as a titer, while other autoantibodies are reported as semi-quantitative index values according to assay methodology.

Test	Result	Reference Range
ANA	1:1280, speckled IFA pattern	<1:80
Anti-Sm	>8*	≤0.9*
Anti-RNP	>8*	≤0.9*
Anti-Ribosomal P	0.5*	≤0.9*
Anti-SSA/Ro	>8*	≤0.9*
Anti-chromatin antibody	>8*	≤0.9*
Anti-dsDNA	2 IU/mL	≤4 = negative, 5-9 = indeterminate, ≥10 = positive
DRVVT (Lupus Anticoagulant) Screening/Confirmatory Ratio	0.87	0.00 - 1.21
Complement C3	72 mg/dL	86-184 mg/dL
Complement C4	6 mg/dL	20-58 mg/dL
ESR	53 mm/hr	0-20 mm/hr
CRP	7.2 mg/dL	≤5.0 mg/dL

Routine testing did not identify leukocytosis, neutrophilic predominance, a major electrolyte abnormality, renal dysfunction, or overt thyroid dysfunction.

Lumbar puncture yielded clear, colorless cerebrospinal fluid with 1 nucleated cell/µL, glucose of 60 mg/dL, mildly elevated protein of 49 mg/dL, and 261 red blood cells/µL. A multiplex CSF polymerase chain reaction panel was negative. The CSF opening pressure was not documented in the records reviewed. Urine toxicology was positive for benzodiazepines after benzodiazepines had been administered in the hospital but was otherwise negative. Recent systemic corticosteroid exposure and uncertain adherence to prescribed risperidone were also considered during the medication review.

A broad autoimmune encephalopathy panel was negative, including antibodies directed against NMDA-R, LGI1, CASPR2, AMPA-R, GABA-B-R, GFAP, amphiphysin, AGNA-1, ANNA-1/2/3, PCA-1/2/Tr, mGluR1, neurochondrin, NIF, and septin-7. The available extracted record did not specify whether this testing was performed in serum, CSF, or both. Anti-ribosomal P antibody was negative. Lupus anticoagulant and beta-2 glycoprotein I IgG, IgM, and IgA antibodies were negative; anticardiolipin antibody results were not identified in the records reviewed, which may limit complete exclusion of thrombotic mechanisms.

Laboratory evaluation prior to rituximab initiation included hepatitis B screening, which demonstrated a nonreactive HBsAg, anti-HBs, and anti-HBc, as well as a negative QuantiFERON-TB Gold Plus test.

Contrast-enhanced brain magnetic resonance imaging demonstrated diffuse dural or pachymeningeal enhancement with mild thickening measuring up to 2 mm. No abnormal intrinsic brain parenchymal signal, restricted diffusion, mass effect, midline shift, intracranial hemorrhage, acute or subacute infarction, or extra-axial collection was identified. The radiology report characterized the finding as nonspecific and included intracranial hypotension and infectious or inflammatory etiologies in the differential diagnosis. The abnormality was therefore considered supportive of a possible inflammatory process but was not regarded as independently diagnostic of NPSLE (Figure 1).

**Figure 1:**
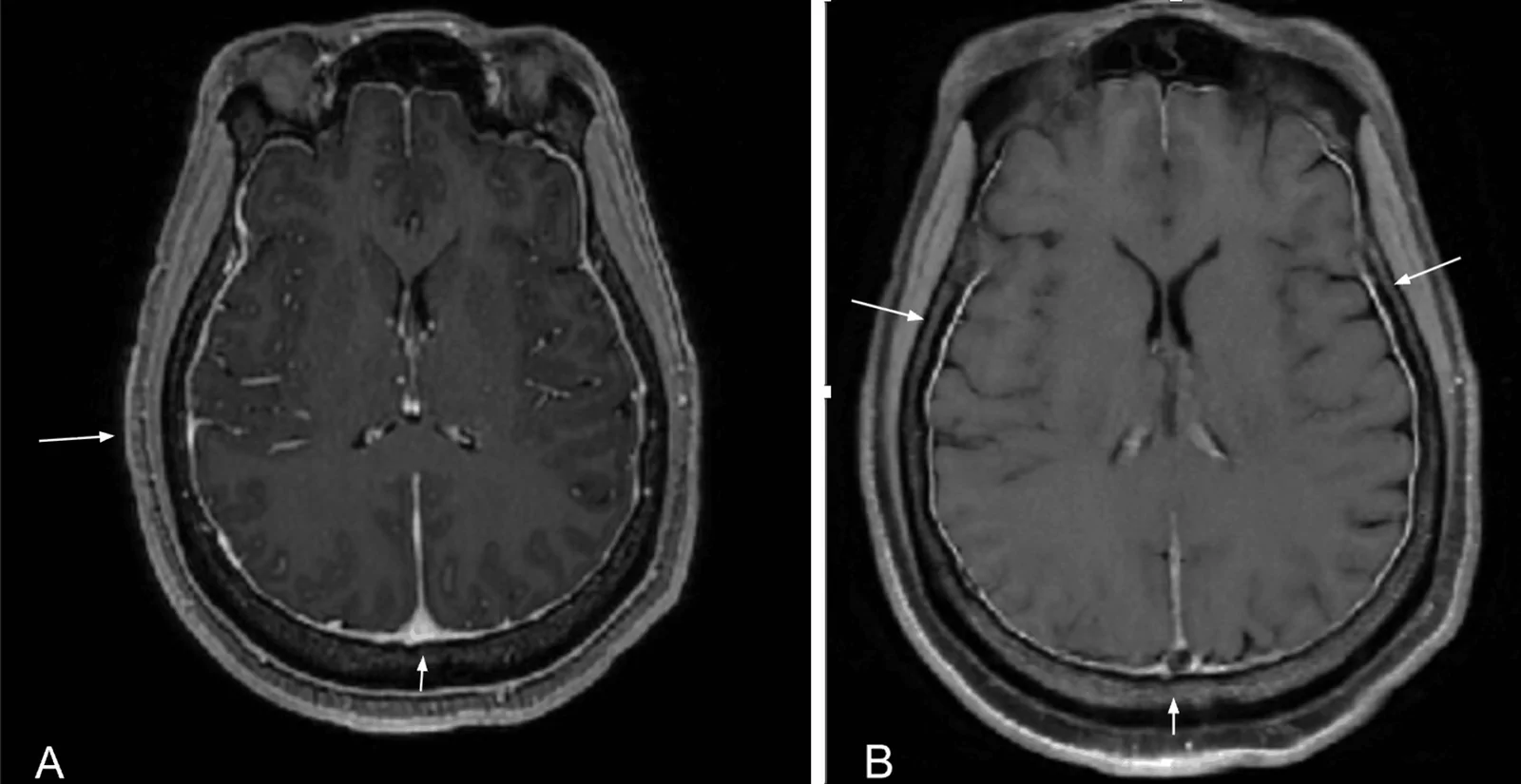
Axial post-contrast MRI brain images Axial post-contrast T1-weighted MRI brain images demonstrating diffuse dural/pachymeningeal enhancement (arrows). These findings are non-specific, but in the clinical context of systemic lupus erythematosus, hypocomplementemia, positive lupus serologies, and exclusion of alternative etiologies raised concern for inflammatory neuropsychiatric involvement. (A) Axial image at the level of the posterior fossa showing dural enhancement along the right temporal convexity and occipital region. (B) Axial image at the level of the cerebral convexities showing bilateral dural enhancement along the frontal and parietal regions.

The multidisciplinary differential diagnosis included a primary psychotic disorder, delirium or metabolic encephalopathy, corticosteroid-induced or corticosteroid-exacerbated psychosis, autoimmune encephalitis, infectious meningitis or encephalitis, steroid-responsive encephalopathy associated with autoimmune thyroid disease, CNS vasculitis or thrombotic disease, substance- or medication-induced psychosis, intracranial hypotension, and other inflammatory or neoplastic causes of pachymeningeal enhancement.

A primary psychotic disorder remained plausible because of the previous schizophrenia diagnosis and psychiatric admission. However, the prior psychiatric episode had also occurred in close proximity to a severe medical illness, and the available records did not demonstrate a clearly established chronic psychotic syndrome independent of medical disease. Delirium was considered because of the initial hypoglycemia, hypotension, inconsistent orientation, and cognitive impairment; these factors may have contributed to the early presentation but did not fully account for the persistence of formal thought disorder and impaired insight after medical stabilization. Recent corticosteroid exposure was also a competing explanation. CNS infection was considered less likely given the absence of fever or meningismus, a CSF nucleated cell count of 1/µL, preserved CSF glucose, and a negative multiplex PCR panel, although the available testing did not exclude every possible infectious cause.

In the context of recently recognized SLE with severe pericardial involvement, hypocomplementemia, strongly positive lupus-associated serologies, persistent psychiatric and cognitive abnormalities, and gradual improvement during lupus-directed treatment, the multidisciplinary working diagnosis was probable NPSLE presenting with psychosis and cognitive dysfunction. Autoimmune encephalitis, medication effects, delirium, and other inflammatory meningeal disorders could not be excluded with complete certainty because portions of the neurological evaluation were unavailable or not performed.

Olanzapine 5 mg twice daily was continued for symptomatic treatment. Rheumatology initiated pulse-dose intravenous methylprednisolone 40 mg twice daily followed by dexamethasone 10 mg daily. Mycophenolate was initiated at 500 mg twice daily and increased to 1,000 mg twice daily. The patient subsequently received one 1-g rituximab infusion. Her psychiatric and cognitive symptoms improved gradually during the hospitalization rather than resolving immediately after a single treatment. She was discharged on olanzapine 15 mg nightly, dexamethasone 10 mg daily, and mycophenolate 1,000 mg twice daily, with continuous family supervision initially recommended. At outpatient rheumatology follow-up one year later, her mental status was documented as having returned to baseline, and she had no clinical signs of active SLE. She was also no longer taking olanzapine and notably had no psychiatric episodes.

Collateral records obtained from an outside institution revealed a prior diagnosis of systemic lupus erythematosus that had not been followed longitudinally. Family members reported that she had been functioning independently prior to her cardiac surgery and had not required psychiatric medications.

Magnetic resonance imaging of the brain demonstrated diffuse dural enhancement with parenchymal thickening, findings concerning for inflammatory involvement (Figure 1). Infectious, metabolic, and structural causes were excluded (Table 2). Neuropsychiatric SLE was considered the leading diagnosis. Rheumatology initiated pulse-dose intravenous methylprednisolone, after which the patient demonstrated marked improvement in psychotic symptoms following two consecutive doses.

**Table 2:** Summary of routine laboratory findings Summary of initial laboratory evaluation demonstrating values within normal limits or at baseline across complete blood count, differential, electrolytes, renal function, and thyroid function studies. These findings do not suggest an infectious, metabolic, or endocrine abnormality that explains the presentation. Findings are presented with corresponding clinical interpretations. BUN: blood urea nitrogen; TSH: thyroid-stimulating hormone

Test	Finding	Values	Reference Range	Interpretation
CBC	WBC/Platelets within normal limits, RBC/Hgb/Hct low Neutrophils within normal limits	WBC 7.45 K/uL	WBC 4.50 - 10.90 K/uL	Not indicative of infection, patient is anemic, hemoglobin was documented to be near baseline
		Platelets 278 K/uL	Plt 130 - 400 K/uL	
		RBC 2.76 K/uL	RBC 4.20 - 5.40 K/uL	
		Hgb 7.8 g/dL	Hgb 12.0 - 16.0 K/uL	
		Hct 25.7%	Hct 37.0% - 47.0%	
Differential	Neutrophils within normal limits, total lymphocytes within normal limits	Neutrophils 55.1%,	38.7 - 60.3%	No neutrophilic predominance
		Neutrophil Abs 3.17 K/uL,	1.51 - 7.30 K/uL	
		Lymphocyte Abs 1.31 K/uL	0.88 - 5.93 K/uL	
Electrolytes	Within normal limits	Sodium 134 mmol/L	136 - 146 mmol/L	No significant metabolic derangement
		Potassium 5.1 mmol/L	3.0 - 5.0 mmol/L	
		Chloride 105 mg/dL	98 - 106 mg/dL	
Renal function	BUN mildly elevated, creatinine within normal limits	BUN 22.0 mg/dL	6.0 - 20.0 mg/dL	Patient does not exhibit significant renal impairment or uremia
		Cr 0.7 mg/dL	0.50 - 0.90 mg/dL	
					Thyroid function	TSH transiently elevated with normal Free T4	TSH 0.900 uIU/mL	0.270 - 4.200 mg/dL	Patient does not exhibit significant thyroid abnormalities
Free T4 1.50 ng/dL	0.93 - 1.70 mg/dL								

This case report was prepared in accordance with the CARE case report guidelines. 

## Discussion

NPSLE is a poorly understood manifestation of SLE with limited literature discussing its prevalence. Reported prevalence estimates vary widely depending on case definition, cohort characteristics, and attribution methodology; estimates include approximately 28.1% in the Swiss SLE cohort and 56.3% in selected prospective/elicited studies from a prior meta-analysis [5,6]. Neuropsychiatric (NP) symptoms frequently occur early in the disease course of SLE. One study evaluating NPSLE patients found that 39% of patients developed NP symptoms within the first year of SLE diagnosis [7]. The American College of Rheumatology has identified 19 distinct neuropsychiatric syndromes attributable to SLE, including cognitive dysfunction, mood disorders, psychosis, seizures, and cerebrovascular events [1,8]. Psychosis is an uncommon but recognized manifestation of NPSLE and may be difficult to distinguish from a primary psychiatric disorder [9]. Similar cases have described patients with NPSLE initially diagnosed with schizophrenia or other primary psychotic disorders before systemic findings led to diagnostic reconsideration [10]. This was similar to our patient, whose prior diagnosis of schizophrenia initially provided a possible explanation for her psychiatric presentation.

NPSLE is a diagnosis of exclusion. Alternative etiologies of NP symptoms, including infection, metabolic derangements, medication effects, structural lesions, and primary psychiatric disorders, must be systematically evaluated before attributing symptoms to autoimmune activity [2]. In this case, the prior history of SLE and recent large pericardial effusion were important clues to an autoimmune etiology. However, the patient's prior SLE diagnosis was documented at an outside institution and was not initially available to the treating team. This fragmented care, combined with her existing diagnosis of schizophrenia, may have contributed to the delayed recognition of NPSLE. The persistence of psychiatric and cognitive abnormalities following medical stabilization, along with positive lupus serologies, hypocomplementemia, and recent pericardial involvement, ultimately raised concern for an autoimmune etiology. Diagnostic uncertainty is further compounded by the limited specificity of neuroimaging findings, which may be normal or nonspecific in patients with NPSLE [1,7]. Patients presenting with mood disturbances, psychosis, seizures, movement disorders, or headaches may initially receive symptomatic therapy directed toward presumed primary neurologic or psychiatric conditions. In milder cases, symptomatic treatment alone may be sufficient. However, in patients with severe or progressive manifestations, additional immunosuppressive or antithrombotic therapy may be required to address underlying inflammatory mechanisms [11]. Delayed recognition of NPSLE may therefore postpone disease-modifying treatment.

Language discordance was a major practical barrier to obtaining a detailed and reliable psychiatric history, but its contribution should be distinguished from the effects of the neuropsychiatric syndrome itself. The record documents assessment by staff without a formal interpreter in the psychiatric emergency setting and use of a medical interpreter during subsequent evaluations. The patient’s husband provided collateral information regarding sleep disturbance, self-directed speech, aggression, failure to recognize her son, medication nonadherence, and changes from her recent baseline; he was not documented as serving as the interpreter. Despite language-concordant support, the patient was frequently minimally engaged, perseverative, disorganized, or nonresponsive and was unable to explain the reason for hospitalization or restate medical information that had just been provided to her.

These circumstances made it difficult to determine whether limited responses reflected language discordance, psychosis, cognitive impairment, delirium, catatonia, or some combination of these factors. Language discordance also limited the precision with which hallucination modality and content, symptom chronology, and subjective mood symptoms could be characterized. The chart does not document specific translation inconsistencies or establish that language discordance independently caused the diagnostic delay. Rather, it increased reliance on family collateral, serial assessments, and outside-record retrieval and compounded the difficulty of evaluating a patient whose attention, memory, insight, and thought organization were themselves impaired. This case therefore supports early use of qualified interpreters or language-concordant clinicians, clear separation of translated patient report from family collateral, and explicit documentation of the limitations of interpreter-mediated mental status examinations [3,4]. It does not support a generalized conclusion that interpreters are unreliable in psychiatric care.

Treatment response must also be interpreted cautiously. The patient received antipsychotic treatment, correction of hypoglycemia and hemodynamic instability, pulse-dose corticosteroids, dexamethasone, mycophenolate, and rituximab during the same hospitalization. Her symptoms improved gradually rather than immediately after a single intervention. Improvement therefore cannot be attributed solely to immunosuppression, and response to treatment should not be considered diagnostic proof of NPSLE. At rheumatology follow-up approximately 10 months later, her mental status was documented as having returned to baseline, and she had no clinical signs of active SLE. This provides meaningful longitudinal evidence of sustained improvement; however, the follow-up was not a structured psychiatric assessment, and the available documentation does not fully characterize the timing of antipsychotic discontinuation, residual symptoms, functional recovery, or risk of psychiatric recurrence.

The clinical significance of this case lies in the interaction between the intrinsic uncertainty of NPSLE attribution and potentially modifiable barriers to diagnostic synthesis. Prior psychiatric diagnoses should be reconsidered when new systemic inflammatory findings emerge, but clinicians should also avoid the converse error of attributing all psychiatric symptoms in a patient with SLE to autoimmune disease. A structured chronology, detailed psychiatric and cognitive examination, medication review, language-concordant assessment, collateral history, outside-record retrieval, and interdisciplinary evaluation are necessary to balance these competing risks.

## Conclusions

This case demonstrates how the intrinsic diagnostic complexity of neuropsychiatric systemic lupus erythematosus (NPSLE) may intersect with contextual and systems-level barriers to prolong diagnostic uncertainty. NPSLE lacks a pathognomonic clinical, serologic, cerebrospinal fluid, or imaging finding and may resemble primary psychosis, delirium, medication effects, autoimmune encephalitis, or other neurologic and inflammatory disorders. Accordingly, the patient’s existing chart diagnosis of schizophrenia reasonably influenced the initial differential diagnosis, although continued reliance on that label risked diagnostic anchoring when new systemic findings emerged. Recognition of the relationship among her recent large pericardial effusion, lupus-associated serologies, hypocomplementemia, cognitive and psychiatric abnormalities, and subsequent clinical course ultimately supported a multidisciplinary working diagnosis of probable NPSLE. Fragmented care across institutions and delayed availability of outside records contributed to the difficulty of integrating these findings, but they should not be viewed as the sole explanation for the diagnostic delay. Language discordance may have further complicated the evaluation. The patient was primarily assessed using interpreter services. Her husband separately provided collateral information regarding her premorbid functioning and the onset of sleep disturbance, self-directed speech, aggression, failure to recognize her son, and episodes of leaving the home. Despite language-concordant support, the patient was frequently minimally engaged, disorganized, and unable to explain the reason for hospitalization or restate recently provided medical information. These features made it challenging to distinguish the effects of language discordance from impaired attention, memory, insight, and thought organization. The available documentation therefore supports language discordance as an additional barrier to nuanced psychiatric and cognitive assessment, but it does not establish it as an independent or primary cause of delayed diagnosis.

Several practical lessons emerge from this case. In patients with new or worsening psychiatric symptoms and evolving systemic inflammatory findings, clinicians should reconsider prior psychiatric diagnoses while maintaining a broad differential that includes delirium, medication effects, infection, and autoimmune or neurologic disease. Language-concordant clinicians or professional interpreters should be used for direct assessment, while family members should provide collateral history rather than serve as substitutes for interpretation. Early retrieval of outside records and interdisciplinary review among psychiatry, internal medicine, neurology or neuropsychiatry, and rheumatology may help establish symptom chronology, identify systemic clues, and reduce avoidable delays. These practices cannot eliminate the inherent uncertainty of NPSLE attribution, but they may improve the accuracy, transparency, and timeliness of diagnostic evaluation.
